# Supplementary figures and images for: Treponema pallidum subsp. pallidum with an Artificially impaired TprK antigenic variation system is attenuated in the Rabbit model of syphilis
Source: PLoS Pathog. 2023 Mar 20;19(3):e1011259. doi: 10.1371/journal.ppat.1011259 (PMC10063172; doi:10.1371/journal.ppat.1011259)

Aaseq

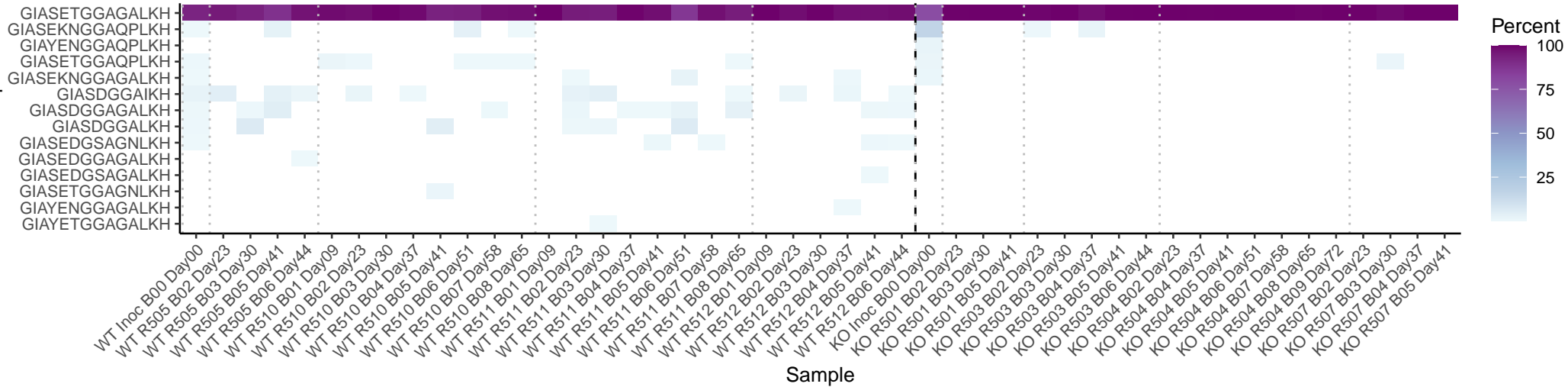

Supplement: S1 Fig — Deep sequencing of TprK V1 region showing persistence of inoculum variants and generation of non-inoculum variants in samples collected overtime from rabbits infected with the WT SS14 strain (“WT”-labeled samples, left side of the map) and form rabbits infected with the SS14-DCKO strain (“KO”-labeled samples, right side of the map), separated by a bold vertical dashed line. In each map, inoculum sequences for the WT strain are labeled as “WT Inoc B00 Day 00”, where WT Inoc indicates WT inoculum treponemes, B00 indicates that the sample was not obtained from a biopsy, and Day 00 indicates the experiment’s time 0. The same nomenclature was adopted for the SS14-DCKO inoculum, with the exception that KO replaced WT. Samples collected post-inoculation report rabbit number (R#), biopsy number (B#), and day post-inoculation the sample was obtained (Day#). Light gray dashed lines separate individual animals. Missing samples did not yield data. The same heatmap in interactive format is available at https://github.com/greninger-lab/Impaired-TprK-Antigenic-Variation. Peptide sequences and prevalence are also reported in S2 Table. (PDF) [file ppat.1011259.s001.pdf]

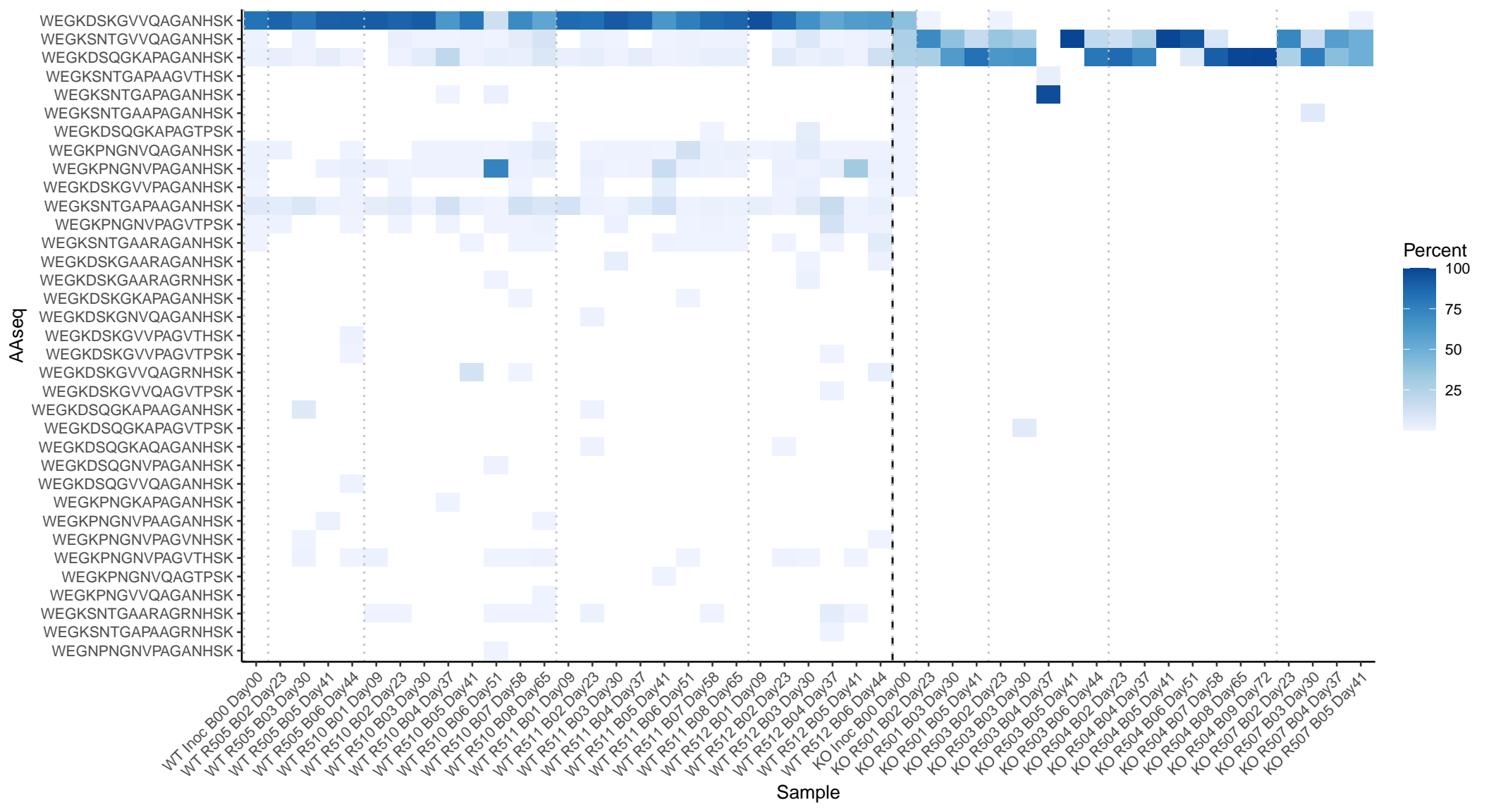

Supplement: S2 Fig — Deep sequencing of TprK V2 region showing persistence of inoculum variants and generation of non-inoculum variants in samples collected overtime from rabbits infected with the WT SS14 strain (“WT”-labeled samples, left side of the map) and form rabbits infected with the SS14-DCKO strain (“KO”-labeled samples, right side of the map), separated by a bold vertical dashed line. In each map, inoculum sequences for the WT strain are labeled as “WT Inoc B00 Day 00”, where WT Inoc indicates WT inoculum treponemes, B00 indicates that the sample was not obtained from a biopsy, and Day 00 indicates the experiment’s time 0. The same nomenclature was adopted for the SS14-DCKO inoculum, with the exception that KO replaced WT. Samples collected post-inoculation report rabbit number (R#), biopsy number (B#), and day post-inoculation the sample was obtained (Day#). Light gray dashed lines separate individual animals. Missing samples did not yield data. The same heatmap in interactive format is available at https://github.com/greninger-lab/Impaired-TprK-Antigenic-Variation. Peptide sequences and prevalence are also reported in S2 Table. (PDF) [file ppat.1011259.s002.pdf]

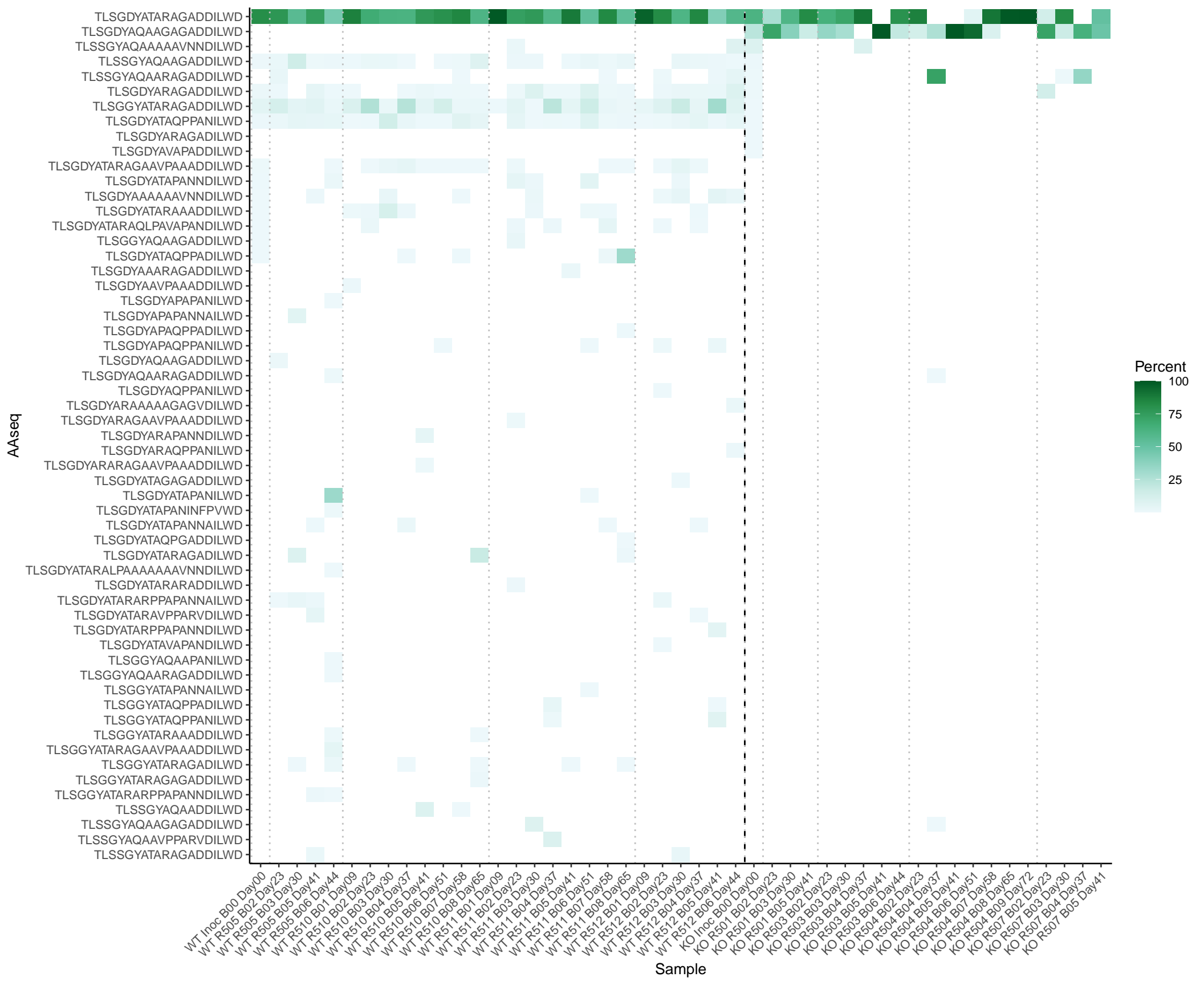

Supplement: S3 Fig — Deep sequencing of TprK V3 region showing persistence of inoculum variants and generation of non-inoculum variants in samples collected overtime from rabbits infected with the WT SS14 strain (“WT”-labeled samples, left side of the map) and form rabbits infected with the SS14-DCKO strain (“KO”-labeled samples, right side of the map), separated by a bold vertical dashed line. In each map, inoculum sequences for the WT strain are labeled as “WT Inoc B00 Day 00”, where WT Inoc indicates WT inoculum treponemes, B00 indicates that the sample was not obtained from a biopsy, and Day 00 indicates the experiment’s time 0. The same nomenclature was adopted for the SS14-DCKO inoculum, with the exception that KO replaced WT. Samples collected post-inoculation report rabbit number (R#), biopsy number (B#), and day post-inoculation the sample was obtained (Day#). Light gray dashed lines separate individual animals. Missing samples did not yield data. The same heatmap in interactive format is available at https://github.com/greninger-lab/Impaired-TprK-Antigenic-Variation. Peptide sequences and prevalence are also reported in S2 Table. (PDF) [file ppat.1011259.s003.pdf]

Aaseq

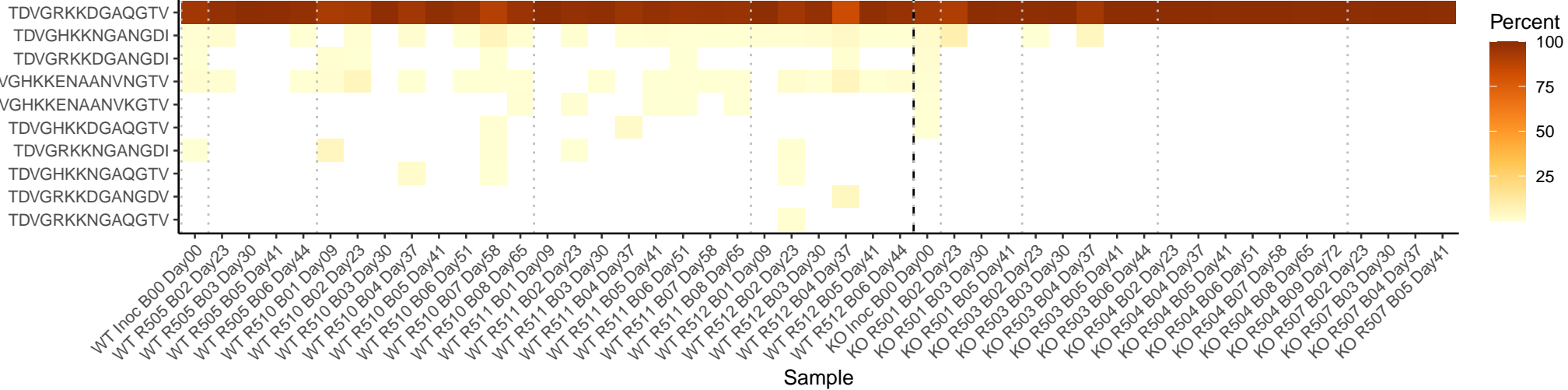

Supplement: S4 Fig — Deep sequencing of TprK V4 region showing persistence of inoculum variants and generation of non-inoculum variants in samples collected overtime from rabbits infected with the WT SS14 strain (“WT”-labeled samples, left side of the map) and form rabbits infected with the SS14-DCKO strain (“KO”-labeled samples, right side of the map), separated by a bold vertical dashed line. In each map, inoculum sequences for the WT strain are labeled as “WT Inoc B00 Day 00”, where WT Inoc indicates WT inoculum treponemes, B00 indicates that the sample was not obtained from a biopsy, and Day 00 indicates the experiment’s time 0. The same nomenclature was adopted for the SS14-DCKO inoculum, with the exception that KO replaced WT. Samples collected post-inoculation report rabbit number (R#), biopsy number (B#), and day post-inoculation the sample was obtained (Day#). Light gray dashed lines separate individual animals. Missing samples did not yield data. The same heatmap in interactive format is available at https://github.com/greninger-lab/Impaired-TprK-Antigenic-Variation. Peptide sequences and prevalence are also reported in S2 Table. (PDF) [file ppat.1011259.s004.pdf]

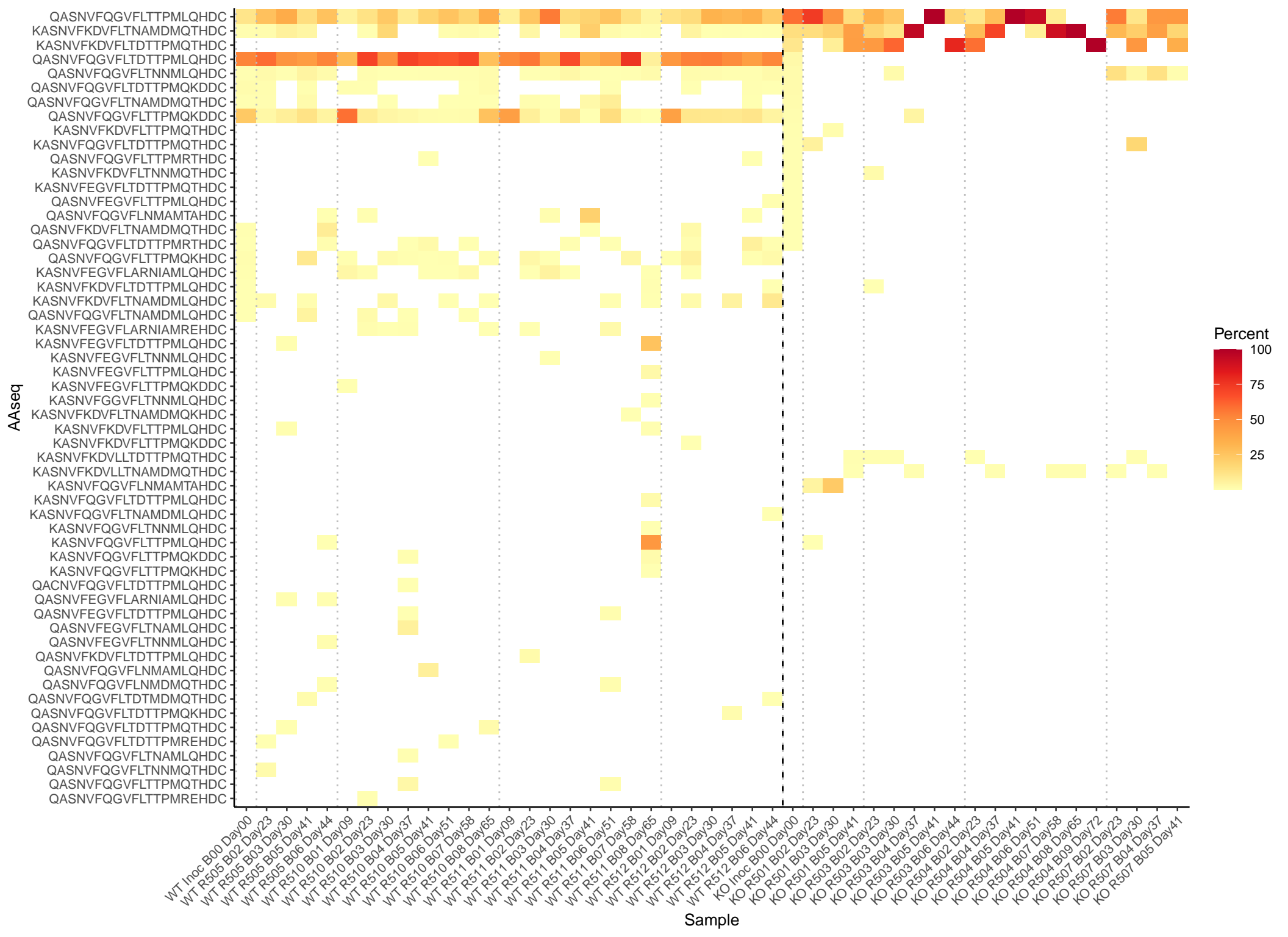

Supplement: S5 Fig — Deep sequencing of TprK V5 region showing persistence of inoculum variants and generation of non-inoculum variants in samples collected overtime from rabbits infected with the WT SS14 strain (“WT”-labeled samples, left side of the map) and form rabbits infected with the SS14-DCKO strain (“KO”-labeled samples, right side of the map), separated by a bold vertical dashed line. In each map, inoculum sequences for the WT strain are labeled as “WT Inoc B00 Day 00”, where WT Inoc indicates WT inoculum treponemes, B00 indicates that the sample was not obtained from a biopsy, and Day 00 indicates the experiment’s time 0. The same nomenclature was adopted for the SS14-DCKO inoculum, with the exception that KO replaced WT. Samples collected post-inoculation report rabbit number (R#), biopsy number (B#), and day post-inoculation the sample was obtained (Day#). Light gray dashed lines separate individual animals. Missing samples did not yield data. The same heatmap in interactive format is available at https://github.com/greninger-lab/Impaired-TprK-Antigenic-Variation. Peptide sequences and prevalence are also reported in S2 Table. (PDF) [file ppat.1011259.s005.pdf]

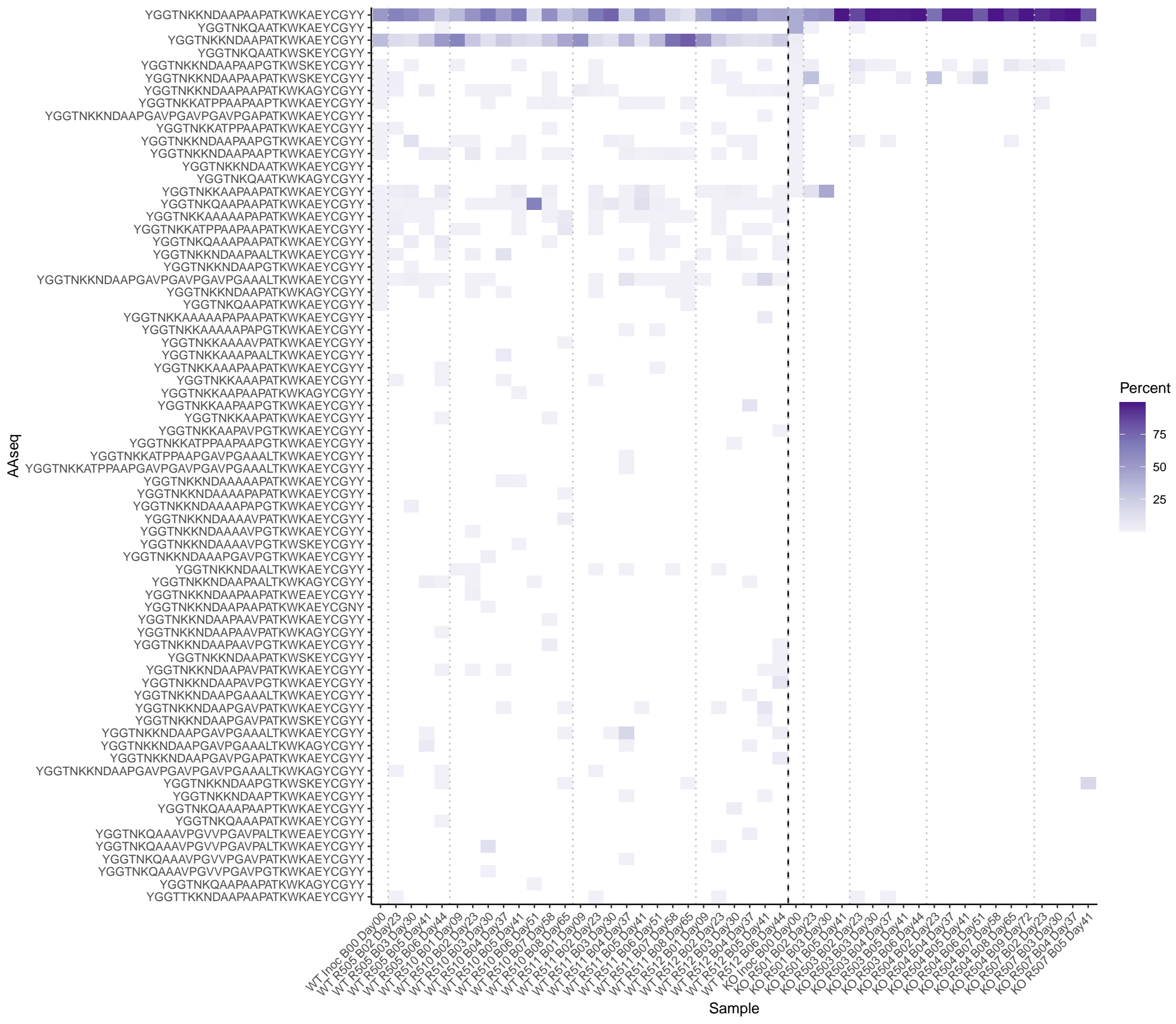

Supplement: S6 Fig — Deep sequencing of TprK V7 region showing persistence of inoculum variants and generation of non-inoculum variants in samples collected overtime from rabbits infected with the WT SS14 strain (“WT”-labeled samples, left side of the map) and form rabbits infected with the SS14-DCKO strain (“KO”-labeled samples, right side of the map), separated by a bold vertical dashed line. In each map, inoculum sequences for the WT strain are labeled as “WT Inoc B00 Day 00”, where WT Inoc indicates WT inoculum treponemes, B00 indicates that the sample was not obtained from a biopsy, and Day 00 indicates the experiment’s time 0. The same nomenclature was adopted for the SS14-DCKO inoculum, with the exception that KO replaced WT. Samples collected post-inoculation report rabbit number (R#), biopsy number (B#), and day post-inoculation the sample was obtained (Day#). Light gray dashed lines separate individual animals. Missing samples did not yield data. The same heatmap in interactive format is available at https://github.com/greninger-lab/Impaired-TprK-Antigenic-Variation. Peptide sequences and prevalence are also reported in S2 Table. (PDF) [file ppat.1011259.s006.pdf]
