# Supplementary material for: Treponema pallidum subsp. pallidum with an Artificially impaired TprK antigenic variation system is attenuated in the Rabbit model of syphilis
Source: PLoS Pathog. 2023 Mar 20;19(3):e1011259. doi: 10.1371/journal.ppat.1011259 (PMC10063172; doi:10.1371/journal.ppat.1011259)
Supplement: S1 File — (DOCX) [file ppat.1011259.s007.docx]

**Insert of the p*DC*arms-47p-*kan*^R^ vector (pUC57 vector backbone)**

**LEGEND**

In aqua = regions upstream and downstream of homology arms (**NOT** **cloned** into vector)

In yellow= homology arms (**cloned** into vector)

In green= *tp0574* promoter. In bold are -10, -35 and Ribosomal Binding Site (**cloned i**nto vector)

In gray = kanamycin resistance gene (**cloned** into vector)

Sense Primers are underlined

Antisense primers are double underlined

TGAAACACAGGCGCTCCGTTCCGTCTGTGCGCCGTGTGCGATACAGTGAGCCTTGATTCTGCGTTTGAAA

GCAGGCACAATGCGTCCCGTGCAGCGTATCATATCTTGGAATGTGAATGGAATTCGTGCCATAGAGCGGA

AAGATTTTCTCAGCTGGCTCGCGCGTGAGGCGCCTGATGTTCTCTGTTTGCAGGAGATTAAAGCGCATGA

GTCGCAGCTGAGTGCTGCGCTTCGTGCTCCGGTCTGGAGTGCTGGGGCGGGGGGTACGTACTATACCTAT

TTTCACAGTGCGCAGCGTCCTGGATACAGTGGCACGGCGCTGTTCAGTAAGCGCGCGCCAGATGCGGTGC

GTTTCTTCGGGGTTCCGGCTTTTGACTGCGAGGGGCGGATGCTTGCGGCACGCTTTGGCGAGCTGACGGT

GGTAAGCGCGTATTTTCCGAATGCGCAGGAAGGGGGCAAGCGGCTCGCGTATAAGCTTGATTTTTGCGCA

GCGTTTCGTGCGTTCTGTGATGAAGAGCGTACGGCCGGGCAGCACGTGATCTTGTGTGGTGACTACAACA

TAGCGCATAAGGAAATCGACCTGGCACATCCTCAGGAAAATGAGGGGAATCCTGGATTCCTGCCTCAGGA

GCGTGCATGGATGGATACATTTACGGAGGCAGGCTATGCGGATAGCTTCCGAGCCTTCTGCACAGAAGGG

CAGCAGTACACGTGGTGGAGCTACCGTGCCCGTGCACGCGCGCGTAACATTGGATGGCGCATCGATTACC

AGTGTGTGGACCAAGCCTTTTTAGCGCGCGTGACCTCTTCGCAGATACTGTCCGAGGTGACAGGATCGGA

TCACTGCCCAGTGTGTTTGACGTACGCGGACTAATCCGTTTCCGGGGTGAGCGGCACGTCCGCGCAAACT

AAGACGTACCCGCGCGCACAGGCAGCGTCAGAGGTGGTAGCGAACGTCCACACCCGCGGCTATGAACTGT

GCGGTGCGCGTGTTGGTCTGCTGTCTATCTTCTTCAATAATCTTTTCGCATGACCGGGGTACGCCGCTGT

ACGTGGCGCTTACCCCCAAGGACCAGTGCTCTGTCAGTTGAAAATAGCACCCCGCTGCCGCCTTGAGCAC

AAGACCGTAGTAGGTAGACGTGTAGTAATGCTGATAATTGAAGCCAGCCCCTACCGTCAGTGGCAAGCGG

ATGCGCCAGAAGGCAACCGTGTACCCGGCAGTGAGGGCAACGGGAATTGCAAGGTAATAGTACGGAGTAG

TGGGACTGTACGTATTGTTTGGATAGCTGCAATGGTACTGCACACTTGCGTCAATCCCGAGCGACAGGCC

GCGGCACACAAAGTGTTCAAACCCTAACGCCGCACTGAACGCGGGGTAGATGTACTTGTGCCCGTTGGTT

TGCGCGTTGGCATTACGGTCGTCCCCGCGCCCGCTGTTACACCAATCCACTTGAAAGAGGGGCACCGCGC

CCATGGCCGAAAGGCGTATAGTACTCCGGCCCGCCGCAGTAGTGTCCCACGGGTCCGCGTGCACCGGGTG

TGCAGCTCCCCACACTCCCGCGCATATTCCCAGCACCGGGCCGACCGCCCACCACTTCAATTGTTTCATA

CCCCGCTCCAACGCCGATCCTCTTACGCGTCTCGTCGAGGACCTACTCCATTCTACCCCCCCCCCACGGC

TGTTTGTCGAACCCTTTTTAAAGGGTTCGTTCTCGCGCGCTGGGCAGCACGCGCGTGAGGCGCCTATGCC

ATCGGGAGCTAGCGGATCCTCCCAAAAAGAGGAAGGACGCGCCTGTGTGTGCTCTGCATAAG

ACG**TTGACA**ATCCCTGTGGGGCGT**GCCTATACT**CAGGCCCTCTATAC**GGAG**GTGTAATC ATGAGCCATATTCAACGGGAGACGTCTTGCTCGAGGCCGCGATTAAATTCCAACCTGGATGCTGATTTAT

ATGGGTATAGATGGGCTCGCGATAATGTCGGGCAATCAGGTGCGACAATCTATCGATTGTATGGGAAGCC

CGATGCGCCAGAGTTGTTTCTGAAACATGGCAAAGGTAGCGTTGCCAATGATGTTACAGATGAGATGGTC

AGACTAAACTGGCTGACGGCATTTATGCCTCTTCCGACCATCAAGCATTTTATCCGTACTCCTGATGATG

CATGGTTACTCACCACTGCGATCCCCGGGAAAACAGCATTCCAGGTATTAGAAGAATATCCTGATTCAGG

TGAAAATATTGTTGATGCGCTGGCAGCGTTCCTGCGCCGGTTGCATTCGATTCCTGTTTGTAATTGTCCT

TTTAACAGCGATCGCGTATTTCGTCTCACTCAGGCGCAATCACGAATGAATAACGGTTTGGTTGATGCGA

GTGATTTTGATGACGAGCGTAATGGCTGGCCTGTTGAACAAGTCTGGAAAGAAATGCATAAGCTTTTGCC

ATTCTCACCGGATTCAGTCGTCACTCATGGTGATTTCTCACTTGATAACCTTATTTTTGACGAGGGGAAA

TTAATAGGTTGTATTGATGTTGGACGAGTCGGAATCGCAGACCGATACCAGGATCTTGCCATCCTATGGA

ACTGCCTCGGTGAATTTTCACCTTCATTACAGAAACGGTTTTTTTATAAATATGGCATTGATAATCCTGA

TATGAATAAATTGCAGTTTCATTTGATGCTCGATGAGTTTTTCTGA

TTACCATGTCACTTTCATTCCGCAGACAAGGGTGCCGAAGTGGCGTTCGGACCAGATGCTCTCGGCAATGCCCATGTAAGGAGCGTCAGCAAGCACGCCCTGTTCCCACTGGGCGCTGAGCTCCACCTTCTCGAAGGGACTGAACGTCAGTCCCACCTGGTACTGGAGCGCGTGCTCACGCAGGAGCGCATCCCCGCTCTGGTTGTGGTTGAAGCGGTTTGTTGCCCATAGCACGGATGTGTGTGGTGCAAGCCAGGCGTGGGAACCGAGGGGGATGCGATAGCTGCACCACGCTTTGCTCAAGATAGGCGTGTTGATGACGCCGTCCGAATTACTTCCCTTGTACTGCGCACCTCCGTTATTTATGTAAAAGATGTAGGTGAGGGGGATGTACACGCGTGCTTCGACGCCGGCGTTCAGGCCGGTGAGCAGGTGGGTGTAGGGGTCACCGCTTTTGGTTTCGAGTTTGAGGAATCCGGCAAAATCAAAGTGATCTGCTTGATTCTTAAAGAAAACACGTTCTCCAAAGATATTAGTGCCTGCGGTGGCAAAGTATATGCCAGAAGAGAGCCACCTCCACTGCATACGCAGGAGCGCGTCTATGTTGAGCGTTTTGGTGTACGTGAATTGCAACCAGGAAATAAAGAGTGCTGTCCGCATCCGCGCGTCAGGATTCTTCTGCTGCTCTATGAGCCTCATGTTTATTTCTAGGCCTTTGCCATTCAGATGTCTTAGGTAACCACCGCTTGACGCTAAGCCGACGATGGTGCGCTGCGCCAAGAGCGTTACTAAGCCAGCTTTCTCCAGAAGACTGCCTTTGCTCAGTTCTTGCACAAAGAATGAAACAACATCTTGTTGCGGGGAGGTCAGCAGCGTGTCCCCAAGACTAGTGATGACTTCATGGACCCTCTGTGAGGGAGCCATTCTAGCGTAGAATTGTGCGTTACTCTGGTGTTGGTTACCGG

CGTCGAGGGCGAAGGAGAAGCGGAAGCCGGCGCCTGGTTCGAGGGTGAGTCGGCCTCCTACTCCCCACAG

GAGTGCTGTTTTGTTTTCGTTCTTGGAGTCTTCGGTACCCTTAACGTAGTTCTGGTCCAGTGTGGCATTC

CCTGCCAGCTCCAACGTAAGCAGCCGCTGACGGTCGACGCCATAGGAAAGCGTTGCATCGGCCCCGAAGC

CATACTTGCTGTGCGTGGTGTCAGTACTATCCCAGGCACCATTGGAAAGGAAGGAGAGGAAACCGATGTC

CACATCTACTCCGCTGTTTCCCACATTGTGGGCCTGGTAGCCGAGTTTTGCCCCGGAGCCGGAGAAACCA

GGGGCATAGCGAGTGTCCTTTTCTGAATAGGCACGGGTGACAAAGGGTTTCCACAGCTGGGCAAAGTTAA

Green sequence primers target the *tp47* promoter.

SENSE AGCGGATCCTCCCAAAAAGA

ANTISENSE GATTACACCTCCGTATAGAG

Blue seqeucne primers target *T. pallidum* DNA regions flanking the DCs homology arms.

SENSE CACAGAAGGGCAGCAGTACA

ANTISENSE CTTCTCCTTCGCCCTCGAC

Gray seqeunce primers: target the *kan*^R^ gene.

SENSE GAGCCATATTCAACGGGAGA

ANTISENSE ATTCCGACTCGTCCAACATC
