# Supplementary material for: Treponema pallidum subsp. pallidum with an Artificially impaired TprK antigenic variation system is attenuated in the Rabbit model of syphilis
Source: PLoS Pathog. 2023 Mar 20;19(3):e1011259. doi: 10.1371/journal.ppat.1011259 (PMC10063172; doi:10.1371/journal.ppat.1011259)
Supplement: Attachment — Submitted filename: Response to reviewers.docx [file ppat.1011259.s012.docx]

**RESPONSE TO REVIEWERS**

**Part I - Summary**

Reviewer #1: In their submitted manuscript, Giancani and colleagues build on their recently developed technological advance – genetic manipulation of Treponema pallidum. In prior studies, this same group described allelic replacement of the tprA locus with a kanamycin resistance marker using a suicide vector. This advance was made possible by the recent development of a system for cultivation of T pallidum in vitro by Norris and colleagues. In their first studies, the Giacani group opted to start with tprA because the corresponding gene product is non-essential. In the current manuscript, the authors extend their prior studies by attempting to knock out tprK in the reference strain SS14. Numerous prior studies by Giacani and others suggest that antigenic variation by TprK promotes immune evasion during natural infection. Although unsuccessful in knocking out tprK, the authors were able to eliminate most of. The cassettes used by tprK for antigenic switching. The resulting SS14-DC_ko strain grew normally in rabbit testes and could be sustained through in vitro passaging but was unable to produce cutaneous lesions on the backs of rabbits inoculated intradermally. Rabbits infected with the DC_ko also failed to seroconvert, further highlighting the importance of TprK antigenic switching for persistence.

Overall, the manuscript is well written, and the authors conclusions are largely supported by the data. The development of a facile system for generating mutants in T pallidum is a major advance for the field in general. The workflow used by Giacani and colleagues to characterize the DC-ko mutant is very through, as would be expected of this group. While the work presented in the submitted manuscript are a major accomplishment. there are some concerns, outlined below, that need to be addressed.

**A: We are grateful for the words of praise from this colleague.**

Reviewer #2: This manuscript by Romeis et al. provides evidence for the importance of tprK sequence variation in the ability of Treponema pallidum to cause persistent infection in mammalian hosts. Deletion of a major portion of the donor sites for tprK variation led to reduced T. pallidum burden, lesion development, and tprK sequence variation in a rabbit model. Importantly, this represents for the first report of a functional mutation in T. pallidum. Limitations include the use of a heterogeneous strain as the starting point and the lack of complementation and thus fulfillment of ‘molecular Koch’s postulates’; the manuscript should be modified to further recognize these aspects. Nevertheless, the manuscript represents a significant step in the study of T. pallidum pathogenesis.

**A: We further highlighted such limitations (lack of an isogenic strain and of a complementation system) in Discussion of the revised manuscript.**

Reviewer #3: The present study by Romais et al sets out to capitalize on a recently developed tool to genetically manipulate the causative agent of syphilis, T. pallidum. Utilizing this tool, the authors successfully deleted 96% of the donor cassettes that provide variation to the tprK gene. Their results demonstrate that the genetically altered T. pallidum SS14 strain is impaired in its ability to vary the TprK antigen, and exhibits attenuated proliferation and disease during infection of rabbits compared to the wild type strain. This work represents the first of its kind on TprK antigenic variation and has the potential to significantly advance our knowledge on the importance of TprK for T. pallidum virulence and persistence. However, the study suffers from a few key weaknesses, most notably the absence of any attempt to complement the observed phenotypes.

**A: We agree and acknowledge in the manuscript that the current lack of a complementation system for *T. pallidum* is a limitation. One of the reviewers mentioned that genetic engineering for *T. pallidum* is “just coming of age”. Given its status, however, it seems more appropriate to say it is in its very early infancy. Tools such as shuttle vectors and transposons are still unavailable to us, even though not for lack of trying. Regarding shuttle vectors, pCF693-based constructs were not retained by *T. pallidum*. Such an initial failure was not unexpected, as no plasmids were ever confirmed for the syphilis agent despite one unsubstantiated report in the early ‘80s (PMID: 6264606). Regarding transposon mutagenesis, transformations with himar-based constructs modified with different *T. pallidum*-specific promoters were also unsuccessful so far in our lab. Upon acquiring the necessary support, the development of these tools will be a priority.**

Reviewer #1: TprK variants in Table 3 indicate that the wildtype and DC-KO are non-clonal. Did the authors isolate only a single DC_ko isolate? Were attempts made to perform limiting dilution to get clonal isolates? The authors indicate that the genomes of the strains used for infection studies were sequenced, but only provide data for tprK. In the absence of complementation. A more thorough analysis of SNPs between the two strains used for IT and ID inoculation is warranted.

**A: We have performed a genome-wide analysis of the sequencing data. Our results show that when the mutant strain and the WT strain are compared, no additional mutations/SNPs that could explain the attenuated phenotype of the DCKO mutant were found. We report these results in the text of the manuscript. Because this analysis yielded negative results, we did not deem it necessary to add any additional figure or table to the manuscript. As already reported, however, the sequencing data are available to all readers in NCBI under** **BioProject PRJNA909291.**

**We had already mentioned in Discussion the importance of repeating this study with a clonal strain of the mutant. In this study, however, we opted to use a near-clonal isolate to evaluate expansion of underrepresented sequences for the V regions. This choice is at the same time a limitation and a strength. In future experiments, we will use limiting dilution of the mutant population to derive a fully isogenic strain in the *tprK* V regions and infect immunocompetent controls and immunosuppressed (Methylprednisolone-treated) animals. These studies should show that the DCKO strain ability to proliferate *in vivo* is fully or partially rescued by immunosuppression. These experiments, however, are costly and require at least one solid year to be performed. As such, they are ideal future directions for the work presented here.**

Reviewer #3: Although the authors have done a commendable job validating the SS14-DCKO mutant clone that includes confirming the singular presence of the integrated kanR gene, the study suffers from the absence of a complement clone for use in infection assays. Understandably, genetic manipulation is only just coming of age for Tp, but the need for some type of complementation to ensure that all mutation-related phenotypes are solely due to loss DCs is especially important given that this is the first time this region has been genetically manipulated. In lieu of a genetic complement, the RIF experiments should at least be carried out in immune-deficient or immune-suppressed rabbits to verify that they behave similar to WT in the absence of a host antibody response. Propagation of the mutant in the immune-privileged testis is not sufficient because it does not involve the same infection processes as those in the RIF experiments.

**A: Please, see the answer above for infection of immunosuppressed animals. RIF is not an acronym used in this manuscript. We assume the referee means RIT. If so, we respectfully believe that it makes sense to perform those in immunocompetent animals receiving lymph node extracts from immunosuppressed or immunocompetent animals previously infected with the mutant or the control strain. Those experiments are ideal future directions for the work presented here. We addressed the criticism concerning the testes as a partially immune-privileged organ in Part III of this document and in the manuscript text.**

In line with the above comment, have the authors looked into the possibility of polar effects on genes flanking the deleted DC regions? Again, this seems warranted to ensure that all observed mutant phenotypes are due to absence of the deleted DCs.

**A: This is an excellent point, and we apologize for not having looked at this already. We have now looked at possible changes in transcription in the *tp0131* gene (immediately downstream of the inserted resistance cassette) in comparison to the WT. We have included these data requested. No transcriptional polar effects were detected by message quantification of the *tprD* gene following reverse transcription with either random hexamers or a gene-specific primer. Quantification of the *kan*^R^ message was used as control and found to be not dissimilar form our previously published work on genetic engineering of *T. pallidum* where the same resistance cassette was used. All messages were normalized to the transcript of the *tp0574* gene.**

Pgs. 12-13, lines 244-246 & 277-280 – The detection of Tp DNA via qPCR appears to contradict the treponemal clearance observed with the RIT. This would seem to suggest that the qPCR only detected residual gDNA from dead treponeme cells. Did the authors attempt to culture Tp recovered from the biopsies and aspirates to determine viability?

**A: Residual gDNA is a likely possibility, particularly given the result of the RIT assay and *tprK* profiling, with the latter being not possible for most DCKO-infected rabbits past day 30 post-inoculation. We mention this in the manuscript more clearly. Unfortunately, given the severe level of attenuation of these lesions (due to the significantly reduced or absent treponemal burden), we opted to save samples for DNA extraction only and not split them for RNA isolation or cultivation. Future experiments, however, will have these components included.**

**Part III – Minor Issues: Editorial and Data Presentation Modifications**

Reviewer #1: The authors noted a difference in lesion development between wt and DC_ko isolates as early as 9 days post-inoculation, well before detectable antibody titers for TprK were observed. Moreover, the authors noted the relatively low levels of expression for tprK. These data seem to argue against Ab-mediated clearance as being responsible for this difference.

**A: We never stated in the manuscript that treponemal clearance was exclusively due to anti-tprK antibodies, but simply that these antibodies might participate in clearance. In the last paragraph of the Discussion, however, we tried to coalesce the molecular and immunological data presented here to provide a working model for our experimental observations. This model will be further validated in the future but will also require a more thorough analysis of the humoral response generated to TprK other V regions, as well as to other putative OMPs, to shed light on why clearance occurs so early. The fact that a low and slow antibody response is generated to TprK in DCKO-infected animals is very likely due to the significantly reduced antigenic (i.e. treponemal) burden in these animals which is often ~3 orders of magnitude lower than in control animals. This poor response inevitably complicates detection of antibodies to single antigens. Even when TPPA assays (that use whole *T. pallidum* cell lysate as antigen) are performed (Fig.5), it is evident that a lower response is mounted against the pathogen in rabbits infected with the mutant. These factors likely made the antibodies responsible for clearance detectable only at later time points post-infection in ELISA.**

**Regarding the level of *tprK* transcription, *T. pallidum* expresses its few putative OMP-encoding genes, including *tprK,* at a very low level (PMID: 34570834; PMID: 15716460, and Ref. 52). The paucity of surface-exposed antigens in this pathogen is a widely accepted mechanism for immune avoidance, and the low level of *tprK* expression seen here agrees with other studies on this gene. Additionally, although low, there is no difference in the *tprK* transcription level between the WT and the mutant.**

Reviewer #2: 1. It would be useful to present any information regarding the transformation frequency. Were the number of ‘input’ bacteria (i.e. the number of T. pallidum adherent at the time of CaCl + DNA treatment) determined? Were all of the wells consistently positive for outgrowth of KanR T. pallidum, or only a portion (e.g. 2/24 wells) positive? Can the frequency of transformation be estimated through a combination of these data and the time of growth detection?

**A: We concur that any information regarding transformation efficiency would be desirable, but unfortunately, a rigorous determination of this parameter is yet to be performed and was not a goal of this work. The number of input bacteria as defined by the referee (i.e., adherent cells at the moment of adding the suicide vector) was not determined. Furthermore, given that the post-transformation propagation protocol causes a fraction of treponemes to be lost at any weekly medium exchange or sub-culturing, any attempt to estimate transformation efficiency with the current data would result in an imprecise estimate that, in turn, would probably generate more criticisms than satisfy a reader. We will address this issue in future experiments.**

2. Fig. 5 and accompanying text. qPCR determinations in these kinds of studies are typically normalized to amplifications of a host gene, whereas in this case are presented as “tp0574 copies/reaction”. Ref. 56, which is cited for the procedure, utilized the human beta-globin gene for normalization. The M&M section does not provide any additional information, such as the amount of DNA used. The amount of DNA purified from each sample would be expected to vary widely, based on size of the biopsy, mincing, efficacy of extraction, etc., so some method for normalization is needed.

**A: We normalized these values to input DNA as requested. As already reported in Methods, all biopsies were 4 mm in diameter, and all were collected and processed in the same manner to minimize variables due to the issues mentioned by this referee. Upon performing this normalization, the patterns of treponemal burden overall did not change compared to the previously presented data. The consistency of our method is ensured by the fact that all samples are collected using the same approach, the same transport medium, that all samples are correctly cryopreserved, that the extraction is performed using the same protocol to extract the whole sample and, lastly, those amplifications are performed using the same template volume.**

**Only for the sake of clarity, Ref.56 refers to human patient samples used for WGS of *T. pallidum* strains, and it is quoted just for the amplification protocol of the *T. pallidum*-specific gene. As reported in Ref.56, the amplification of the beta-globin gene from the clinical samples is not used to normalize the treponemal burden but to assess the presence of amplifiable human DNA as an extraction control. To establish whether a sample is suitable for WGS, the only needed information is the copy number of *tp47*/volume of reaction, which is an accurate indicator of treponemal burden in the sample to determine whether a sample is suitable for WGS.**

3. l. 268. Was qPCR performed on the popliteal lymph node samples as another measure of T. pallidum quantity?
**A: Unfortunately, it was not, as the lymph node extract is limited in volume, and it was used in its entirety to inject the RIT recipient. This is, however, an excellent suggestion, and we will save lymph node extract for qPCR in future experiments. Suppose one wanted to use data from Turner and Hollander’s fundamental work “Biology of Spirochetes” that correlate inoculum size and time to orchitis. In that case, one could infer that the inoculum in the lymph node extracts of the control rabbits was between 10 and 100 treponemes, but given that we lack direct quantification data, we prefer to leave out this extrapolation.**

4. l. 322-324. This statement is unclear as written.
**A: The statement was reworded to improve clarity.**

5. The labeling in Fig. 6 is far too small to visualize in the journal format. Also, in this and in similar supplemental figures, the SS14-WT and SS14-KO regions are not labeled. Another way to present this information in the article should be developed; each of the V region datasets could then be included as supplemental figures.
**A: We agree, particularly regarding the inability to discern the sample ID below the X-axis. We, therefore, adopted a new and improved visualization approach for the data and provided only one panel in the manuscript for V6. The other heatmaps are now provided as supplemental material and also as interactive webpages where peptide sequences and rabbit identifiers can be easily seen. We respectfully point out that the scope of these figures, however, is to appreciate the “big picture” related to the generation of non-inoculum TprK variants in the WT (very high) and mutant (practically absent) strains over time. The chosen format, in our opinion, serves this purpose wonderfully. All these data (peptide sequences, rabbit ID, time points, variant prevalence) sequences are also conveniently reported and organized in Table S2.**

6. Table 3. For regions V5 and V6, the predominant genotypes for SS14-WT and SS14-KO. This pattern is also evident in the T. pallidum recovered from infected rabbits (e.g. Fig. 6). A possible explanation is that a subset of clones in the SS14-WT population were mutated. Possible explanations should be provided in the Discussion.

**A: The first sentence appears in need to be completed. SS14-WT has a functional antigenic variation system. Contrary to other spirochetes, we have shown that *tprK* also varies when *T. pallidum* is propagated in vitro (PMID: 34492041). For this reason, it is nearly impossible to maintain a parent strain with its original *tprK* sequence by the time we can attain sufficient cells for animal infection. We addressed the issue of lack of *tprK* isogenicity in the Discussion. As reported above, we have performed a genome-wide analysis of the sequencing data. Our results show that when the mutant and the WT strains are compared, no additional mutations or SNPs that could explain the attenuated phenotype of the DCKO mutant were found. Because this analysis yielded negative results, we did not deem it necessary to add an additional figure or table to the manuscript. As already reported, however, the sequencing data are available in NCBI under BioProject PRJNA909291.**

7. l. 373-376. This statement represents part of the results, and should be moved to the Results section.

**A: This concise statement refers to experiments attempted in the lab prior to targeting the DCs. We needed to mention those because readers would wonder why we targeted the DCs instead of the tprK ORFs. The Discussion section is the appropriate place to mention these failed experiments to introduce the hypothesis that *tprK* might be an essential gene. Therefore, we ask to keep this sentence where it is rather than adding more paragraphs and sections to Methods and Results regarding experiments that failed and yielded no data.**

8. l. 395-398. Only the seminiferous tubules of the testes and associated ducts represent an immune-privileged site. The connective tissue where T. pallidum propagates is not immune-protective, as indicated by the rapid elimination of the bacteria after infiltration of the site by T cells and macrophages. The particular time point that the authors examined apparently was before this infiltration occurred. It is likely that a similar pattern of more rapid elimination of SS14-KO from the testes would have been evident if a time course were performed. The text should be modified to reflect this consideration.

**A: We agree that although the tubules occupy most (80% at least) of the testes volume (PMID:** **10560008), this organ is only partially immune-privileged. We modified the text as requested.**

9. l. 420. Most would argue that not beginning with an isogenic population and isolating the KO mutant from that strain is a true limitation in this study; it complicates the interpretation of the data, as exemplified by tprK sequence variation results. Starting with a clonal population is the paradigm for bacterial genetic studies. It may be best to accept this limitation and state that more definitive studies beginning with a clonal population will be done in the future.

**A: As requested, the text was modified to indicate this overall limitation. We point out, however, that Discussion already reported that “We are nonetheless deriving an SS14-DC^KO^ strain fully isogenic for TprK to evaluate the hypothesis that, upon ID infection with this strain, early disease manifestations will be even more accentuated compared to the present study”. This statement implied already that more definitive studies are being planned.**

10. Another limitation is the lack of complementation and restoration of WT infectivity patterns. Thus it is possible that the observed phenotype is due to other ‘background’ mutations in the recovered SS14-KO strain. This is implied, but should be stated more clearly in the Discussion.
**A: As reported above, we have performed a genome-wide analysis of the sequencing data. Our results show that when the mutant and the WT strains are compared, no additional mutations or SNPs that could explain the attenuated phenotype of the DCKO mutant were found. Because this analysis yielded negative results, we did not deem it necessary to add another figure or table to the manuscript. As already reported, however, the sequencing data are available in NCBI under BioProject PRJNA909291.**

**We agree that the lack of ways to complement a *T. pallidum* mutant is a limitation. Unfortunately, we have not yet been able to overcome it, despite our efforts to transform *T. pallidum* with shuttle vectors.**

11. l.430-443. Interpretation of these results is complicated by the lack of long-term serologic testing in RIT recipients of lymph node extracts from 505 and 512 (Table 2). Also, both recipients receiving the day 79 inoculum seroconverted, contrary to line 432. It is suggested that this section be deleted or modified.

**A: To avoid confusion, we specified which rabbits we are referring to in this. Line 432 refers to RIT rabbits that received lymph node extracts from animals previously infected with the SS14-DC^KO^ strain. These RIT rabbits did not seroconvert. Therefore, the sentence is correct. The interpretation of these results is straightforward and not complicated because all control rabbits fulfilled the criteria for RIT positivity, while all the test animals failed to do so. RIT positivity is defined as either development of DFM-positive orchitis (with or without positive serology) or positive serology (in the absence of orchitis), with the latter situation being more common than the former one. Once one of these criteria for positivity is fulfilled, the animals are euthanized without any need to acquire additional data or to protract the experiment. This is standard practice in all RIT experiments. On the contrary, animals that neither develop orchitis nor seroconvert must be kept in the study for 90 days before being declared syphilis-free. In our specific case, all RIT rabbits that received extracts from the control rabbits developed DFM-positive orchitis, which alone constitutes a diagnosis of syphilis, while none of the RIT recipients of extracts from the test rabbits did.**

12. l. 449. The wording “led to clearance” is an overstatement, in that T cell responses were likely also important. Perhaps ‘likely contributed to clearance’?

**A: The text was modified accordingly.**

13. l. 465. The ‘unable to proliferate’ statement seems contradictory to l. 160-164, and should be modified.

**A: Lines 160-164 refer to the IT infection model, while line 465 in Conclusions refers to the results of the ID infection experiment. We added “ID” to line 465 of the manuscript to reflect this difference.**

14. l. 484. Please indicate whether the rabbits were kept in a low-temperature facility and provided antibiotic-free food and water.
**A: This information is now provided.**

15. None of the supplemental figures or tables have titles, legends or footnotes (except for Table S3). Please provide this information.
**A: Legends for the supplementary figures and Tables were added to the manuscript text.**

Minor

l. 164. Please provide the total number of T. pallidum extracted, rather than T. pallidum/ml (which the reader can’t interpret without knowing the number of mls extracted).

**A: the requested information was added to both line 164 and Methods.**

Figure legends. Each figure legend should start with a title encompassing the overall figure (before A).

**A: Each figure now starts with a title encompassing the overall figure before panel A. unless a single panel constitutes the figure. Legends for supplementary files/figures were added as well.**

l. 214-216. Change figure references to Fig. 3F and Fig. 3G.

**A: Figure references were corrected.**

Fig. 4. The statistical ‘brackets’ used are confusing because they resemble the bars, and should be replaced by a single horizontal line. Also, the “ns” designation for the last timepoints is not referred to in either the legend or the text; it does not seem to be important, and should be removed from the figure.
**A: brackets were changed into lines. Regarding the “ns” designation, we had stated in the submitted manuscript that “In control animals, lesion size steadily increased throughout the experiment (Fig.4A) up to day 65 post-inoculation (*p*>0.05 for lesion diameter values between day 65 and day 72 in WT-infected animals)”. Therefore, we kept the designation, mainly because past day-65 there is still an upward trend in the size of the control lesions that, however, is not significant. The designation “ns” was added to Fig.4 legend for completeness’ sake.**

l. 546-547. old medium … fresh medium.
**A: Media was changed to medium.**

l. 698. Indicate the size of the punch biopsies.
**A: The information was added to the text.**

l. 708. “in case” is incomplete.

A: **The sentence was rephrased**.

Reviewer #3: Pg. 16, lines 324-325 – This statement seems a bit overreaching given that a number of variants are detected in the DC KO mutant. Something to the extent of “significantly decreased” or similar seems more in line with the results.

**A: We replaced “virtually stopped” with “significantly reduced”, although, in the experiments described here, we did not find any variant that was not present in the inoculum.**

Pg. 20, lines 412-414 & 428-430 – It seems unlikely that a highly conserved DC in Tp would play a marginal role in TprK antigenic variation. Other possibilities are more plausible, including that V7 variation may be more important during late-stage infection. Such alternatives should be at least briefly mentioned in the text.

**A: We acknowledged this possibility in the text.**

Pg. 23, line 501 – text should be revised to read “in a previous study”.
**A: The text was revised accordingly.**

Pg. 38, line 838 – revise “form” to “from”.
**A: The text was revised accordingly.**

Throughout the manuscript – need more consistency with the use of WT treponemes vs wild-type treponemes.

**A: We substituted “wild-type” with WT throughout the manuscript with only one exception, not to start a sentence with an acronym, which would be acceptable but not recommended.**
